# Oxidative Stress and Longevity in Okinawa: An Investigation of Blood Lipid Peroxidation and Tocopherol in Okinawan Centenarians

**DOI:** 10.1155/2010/380460

**Published:** 2011-03-30

**Authors:** Makoto Suzuki, D. Craig Willcox, Matthew W. Rosenbaum, Bradley J. Willcox

**Affiliations:** ^1^Okinawa Research Center for Longevity Science, Okinawa 901-2114, Japan; ^2^Department of Human Welfare, Okinawa International University, Okinawa 901-2701, Japan; ^3^Faculty of Medicine, University of the Ryukyus, Okinawa 903-0215, Japan; ^4^Pacific Health Research and Education Institute, Honolulu, HI 96813, USA; ^5^Department of Research, Kuakini Medical Center, Honolulu, HI 96817, USA; ^6^Department of Research, Planning and Development, The Queen's Medical Center, Honolulu, HI 96813, USA; ^7^Department of Geriatric Medicine, John A. Burns School of Medicine, University of Hawaii, HI 96817, USA

## Abstract

*Background*. The Free Radical Theory of Aging mechanistically links oxidative stress to aging. Okinawa has among the world's longest-lived populations but oxidative stress in this population has not been well characterized. *Methods*. We compared plasma lipid peroxide (LPO) and vitamin E—plasma and intracellular tocopherol levels (total *α*, *β*, and *γ*), in centenarians with younger controls. *Results*. Both LPO and vitamin E tocopherols were lower in centenarians, with the exception of intracellular *β*-tocopherol, which was significantly higher in centenarians versus younger controls. There were no significant differences between age groups for tocopherol: cholesterol and tocopherol: LPO ratios. Correlations were found between *α*-Tocopherol and LPO in septuagenarians but not in centenarians. *Conclusions*. The low plasma level of LPO in Okinawan centenarians, compared to younger controls, argues for protection against oxidative stress in the centenarian population and is consistent with the predictions of the Free Radical Theory of Aging. However, the present work does not strongly support a role for vitamin E in this phenomenon. The role of intracellular *β*-tocopherol deserves additional study. More research is needed on the contribution of oxidative stress and antioxidants to human longevity.

## 1. Introduction

Human longevity is a complex phenotype that is determined by environment, genetics, and chance [1]. Understanding the mechanisms by which aging leads to longevity, particularly healthy longevity would be of enormous benefit to our aging population. Unfortunately, most research on human aging has focused on phenomenological description of age-related diseases, and much less is known about the mechanisms of aging itself [2]. Among the most promising theories about how and why we age is the Free Radical Theory, initially proposed by Denham Harman in 1956 [3]. 

Harman proposed that oxygen radicals produced during aerobic respiration induce oxidative damage in DNA, cells, tissues, and organisms that lead to aging and death. Studies in Harman [3] hypothesized, based on observations of enzymatic redox chemistry, that oxygen radical generation occurs *in vivo* and that mechanisms exist to protect against such damage. Mitochondria were later found to be a principal source of these oxygen radicals [4]. The enzyme superoxide dismutase (SOD) provided early evidence for endogenous antioxidant defenses against such radical damage [5]. Exogenous antioxidant defenders have also been discovered in food sources, among the most studied being vitamin E [6, 7]. 

Vitamin E consists of a group of eight related fat soluble tocopherols and tocotrienols, which have significant antioxidant properties [6]. The highest bioavailability, absorption, and metabolization occur with *α*-tocopherol [7]. 

Some research supports *α*-tocopherol as the most important lipid soluble antioxidant since it protects cell membranes from chain reactions caused by radical-induced lipid peroxidation through quenching free radical intermediates [7]. While the antioxidant role of other forms of vitamin E is less clear, *γ*-tocopherol is a nucleophile that can quench electrophilic mutagens, *β*-tocopherol has similar properties, and tocotrienols can protect neurons from oxidative stress [8–10].

Based on this background, we and others have investigated LPO, SOD, and vitamin E as potential factors in human aging and longevity. Plasma LPO has been found to increase with age into the septuagenarian years [11] but little is known about population levels in exceptionally aged individuals, such as centenarians. Studies in Mezzetti et al. [12] found that lower plasma lipid peroxide and higher plasma vitamin E predicted lower risk of cardiovascular events (an important cause of premature mortality) in healthy Italian octogenarians over a 4-year period. Studies in Suzuki et al. [13] found that plasma LPO was lower in Okinawan centenarians than septuagenarians, consistent with their demographic selection against mortality. This is consistent with the hypothesis that protection against oxidative stress might be a survival factor in older Okinawans and possibly a marker of “slower” aging. 

In order to assess potential connections between LPO and common endogenous or exogenous antioxidants, Suzuki et al. [14] studied whether Okinawan centenarians also displayed high levels of plasma or intracellular SOD. Contrary to expectations, SOD levels were lower in centenarians than in younger controls. This might be, in part, a reaction to low levels of oxidative stress in centenarians but it does not offer support for SOD as a major protector against oxidative stress in this population. 

Conversely, vitamin E intake is known to be high in the traditional Okinawan diet due to abundant consumption of foods such as soy beans (tofu and soy bean oils), sweet potatoes, papaya (and its leaves), and green leafy vegetables (e.g., swiss chard, spinach, mustard greens, and numerous other local varieties) [15, 16]. This is reflected in relatively high plasma *α*-tocopherol in older Okinawans, who are more likely to consume a traditional diet [13]. A recent comparative study found that mean *α*-tocopherol levels were similar between Okinawan elderly and healthy elderly from the USA (Oregon) with and without adjustments for total cholesterol or triglycerides [16]. This was despite the fact that few Okinawans consumed vitamin supplements while more than a third of US elders did. *α*-tocopherol is typically the most abundant tocopherol in human serum since it is preferentially conserved by the liver. For example, one study of middle-aged Japanese women found that *α*-tocopherol formed 88% of serum tocopherol, while the reminder consisted of 8.5%  *γ*-tocopherol, 1.8%  *δ*-tocopherol, and 1.7%  *β*-tocopherol [17]. 

Foods also vary markedly in their tocopherol content. For example, soy oil is mainly *γ*-tocopherol (>60%) whereas sunflower oil is mainly *α*-tocopherol (>90%) [17]. Few foods have a significant quantity of *β*-tocopherol [18] but some traditional Okinawan foods, including brown seaweed and hot red peppers, are rich in *β*-tocopherol; soy oil (in tofu, other soy foods and cooking oil), while not as *β*-tocopherol-rich, is consumed in quite high quantity, particularly in the traditional Okinawan diet, which is more likely to be consumed by centenarians [19]. 

As an important fat soluble antioxidant, Vitamin E and/or its various tocopherols could theoretically explain the low LPO in Okinawan centenarians. However, Suzuki et al. [13, 14, 20] showed in prior work that vitamin E, like SOD, was significantly lower in the plasma of centenarians than of younger controls, even after adjustment for lipids and other factors. Nevertheless, vitamin E subtypes (tocopherol subtypes) and/or intracellular tocopherol levels were not studied. Since the major exposure to oxidative stress in humans originates from mitochondria and this stress occurs mainly intracellularly, it is possible that intracellular concentrations of vitamin E (or its subtypes) may be more important than plasma vitamin E. 

Therefore, we studied Okinawan centenarians and younger control populations with the following primary aims: (1) to quantify blood levels of lipid peroxide (LPO), a common proxy measure of oxidative stress; (2) to quantify blood and intracellular levels of vitamin E (measured in red blood cells), a common antioxidant; (3) to assess potential correlations between LPO and vitamin E levels.

## 2. Materials and Methods

This study was conducted under ongoing Institutional Review Board approval from Okinawa International University, and informed consent was received from all study participants. A total of 139 centenarians (30 males, 109 females, mean age = 100.3 years), residing in Okinawa, were previously studied and had banked blood samples [20]. Additional younger controls were recruited for comparison purposes in the current study. A physical examination was performed at the participants' place of residence. Examination included hematological and biochemical analysis of blood, activity of daily living survey, and anthropometric measurements. For the control group, 79 healthy community-dwelling people (34 males, 45 females, aged 20–80, mean age 62.5 years) were recruited and subjected to identical examinations under similar conditions. These controls consisted of younger (twenties, thirties) and older (sixties, seventies, eighties) participants. Total numbers of cases and controls varied according to the particular assay performed. 

Blood samples were taken in EDTA tubes and stored on ice. Samples were then transported to the University of the Ryukyus Hospital, where they were separated into plasma and blood cell components by centrifugation. Ultracentrifugation was used to evaluate plasma total cholesterol and cholesterol subfractions. LPO was measured using the thiobarbituric acid (TBA) reaction for fatty acid oxidation otherwise known as the “TBA method” [21, 22]. For tocopherol studies, samples were frozen on dry ice and sent to Eizai Research Institute, where tocopherol concentrations were measured by high performance liquid chromatography (HPLC) [23]. Cases and controls were compared using Student's *t*-test.

## 3. Results

Table 1 demonstrates that plasma LPO levels of centenarians were 1.49 ± 0.51 nmol/ml in males (*n* = 30), 1.72 ± 1.28 nmol/ml for females (*n* = 109), and 1.67 ± 1.16 nmol/ml overall (*n* = 139). Centenarians had significantly lower LPO levels than control subjects in their twenties (*n* = 8; 3.30 ± 1.25; *P* < .001), thirties (*n* = 16; 3.51 ± 1.08; *P* < .001), seventies (*n* = 29;  3.40 ± 0.79; *P* < .001), and eighties (*n* = 8; 2.91 ± 0.34; *P* < .001). 

Table 2 displays average (mean) plasma tocopherol levels (total and by subtype) across age strata. Total plasma tocopherol (T-Toc) in centenarians was 8.89 ± 2.31 *μ*g/ml for males (*n* = 19) and 11.05 ± 3.41 *μ*g/ml for females (*n* = 91), and 10.58 ± 3.32 for both groups combined (*n* = 110). Centenarian plasma T-tocopherol (males and females combined) was significantly lower than that of every younger age group (*P* < .001). This trend generally held true for both genders as well, with T-tocopherol increasing with age in both genders until the age of 70s and then dropping thereafter. 

We also compared the levels of plasma tocopherol subtypes (*α*-, *β*-, *γ*-tocopherol) in centenarians to younger controls. Plasma *α*-tocopherol in centenarian males (7.79 ± 2.10 *μ*g/ml, *n* = 19), females (9.82 ± 3.35, *n* = 72), and combined total (9.37 ± 3.32 *μ*g/ml, *n* = 91) showed similar trends as T-Toc, with combined plasma *α*-tocopherol level in centenarians significantly lower than in younger age groups across all age strata (significance levels ranged from *P* < .05 to *P* < .001). Comparisons of centenarians across age strata within gender groups were significant between septuagenarians in females and between all age groups in males. This was partly limited by small numbers. For both genders *α*-tocopherol tended to increase with age and peak at septuagenarian years, then dropped rapidly thereafter.

Total plasma *β*-tocopherol levels in centenarian males (0.18 ± 0.07 *μ*g/ml, *n* = 18), females (0.20 ± 0.21 *μ*g/ml, *n* = 69), and gender combined total (0.20 ± 0.19 *μ*g/ml, *n* = 87) were significantly lower than in septuagenarians but not in other age groups (male *P* < .05; female *P* < .01). Similar relations were seen as with *α*-tocopherol where peak values appeared in the septuagenarian years and dropped rapidly thereafter.

Plasma *γ*-tocopherol levels for centenarian males (0.93 ± 0.42 *μ*g/ml, *n* = 18), centenarian females (1.03 ± 0.59 *μ*g/ml, *n* = 72), and both genders combined (1.01 ± 0.52 *μ*g/ml, *n* = 90) were significantly lower than septuagenarians with regard to the combined (*P* < .05) and female (*P* < .05) centenarian groups. Levels tended to peak in the septuagenarian years for the combined group and were less reliable in individual groups, possibly due to small numbers. 

Gender-related differences appeared with significantly higher T-tocopherol (*P* < .05) and *α*-tocopherol (*P* < .01) found in female centenarians compared to male centenarians.

Table 3 demonstrates comparisons between intracellular tocopherol levels (total and by subtype) between centenarians and controls in various age strata. Intracellular T-tocopherol in centenarian red blood cells in males (1.80 ± 1.00 *μ*g/ml, *n* = 19), females (2.30 ± 1.22 *μ*g/ml, *n* = 77), and both sexes combined (2.19 ± 1.19 *μ*g/ml, *n* = 96) was significantly lower than in all other age groups for combined genders versus all individual age strata (*P* < .001). This relation held when separated by gender for most individual age groups. 

Intracellular concentration of individual tocopherol subtypes (*α*-Toco, *β*-Toco, *γ*-Toco) was also compared between age groups by gender. In centenarians, *α*-tocopherol levels in males (1.51 ± 0.84 *μ*g/ml, *n* = 19), females (2.00 ± 1.13 *μ*g/ml, *n* = 77), and both sexes combined (1.89 ± 1.09 *μ*g/ml, *n* = 96) were significantly lower than in all younger age groups when both sexes combined were compared to younger age groups; significance levels ranged from *P* < .05 (octogenarians) to *P* < .001 (septuagenarians). Peak values tended to be reached in octogenarian years in females with no clear pattern in males until a sharp decline in centenarians of both genders. 

Intracellular concentration of *β*-Toco in male centenarians (0.08 ± 0.04 *μ*g/ml, *n* = 10), female centenarians (0.10 ± 0.08 *μ*g/ml, *n* = 37), and both sexes combined (0.09 ± 0.07 *μ*g/ml, *n* = 47) was the highest of all age groups. However, only the difference between centenarians and controls in their seventies was significant (*P* < .05 males; *P* < .01 females; *P* < .01 combined). This was likely due to the small numbers of study participants in individual age groups other than the septuagenarian age group, since concentrations did not appear to be different across age strata until centenarian years. 

Intracellular concentration of *γ*-tocopherol in centenarian males (0.25 ± 0.16 *μ*g/ml, *n* = 18), females (0.23 ± 0.19 *μ*g/ml, *n* = 74), and both sexes combined (0.24 ± 0.18 *μ*g/ml; *n* = 92) appeared lower than all other age groups. However, it reached significance versus only two groups—septuagenarian females (*P* < .05) and septuagenarian sexes combined (*P* < .05). Lack of significant difference between centenarians and other groups may have been influenced by small numbers of participants in comparison groups. 

Table 4 demonstrates T-tocopherol, *α*-tocopherol, and *γ*-tocopherol in centenarians versus a younger control group (septuagenarians) separated by gender and adjusted for plasma total cholesterol (Tc) and HDL cholesterol (HDL-C) by dividing tocopherol levels by cholesterol levels. This comparison was important since differences in tocopherol between age groups might be an artifact of differences in plasma lipid levels that occur commonly with age. Older populations (age 70 years or more) tend to have lower cholesterol levels, which may partly reflect malnutrition [24]. As a fat soluble vitamin, most tocopherol is carried in lipid subfractions and therefore might appear artificially low if not adjusted by plasma lipid levels. Despite adjustment, there were no significant differences between centenarians and septuagenarians across age strata. 

Tables 5 and 6 show tocopherol: LPO ratios in plasma (Table 5) and intracellular (Table 6) compartments across age strata, separated by gender. No significant differences were found for either gender between the centenarians and controls in their seventies. 

Table 7 shows correlation indices between plasma tocopherol/tocopherol subfractions and LPO as well as between intracellular tocopherol and LPO in two age groups: centenarians and septuagenarians. In centenarians, no significant correlations were found between plasma tocopherol nor tocopherol subfractions and LPO. However, in septuagenarians there were some correlations between tocopherol levels and LPO: (1) a weakly positive correlation was found between plasma T-tocopherol and LPO (*r* = .34; *P* < .01), and between *α*-tocopherol and LPO (*r* = .33; *P* < .01); (2) a weakly negative correlation was found between intracellular T-tocopherol and LPO (*r* = −.32; *P* < .05) and between intracellular *α*-tocopherol and LPO (*r* = −.29; *P* < .05).

## 4. Discussion

In 1956 Harman proposed the Free Radical Theory of aging [3]. He hypothesized that aging is partly due to cumulative oxidative damage, and over the last four decades many studies have supported this view. For example, the literature shows that a progressive accumulation of oxidation-derived damage to cellular machinery is linked to senescence [2, 25]. Lipids, the major component of cell membranes, which are in close proximity to mitochondria, the key source of endogenous free radicals, are prime targets for this damage [26]. As humans age this damage can be observed as a progressive increase in blood lipid peroxidation byproducts [26]. Therefore, lipid peroxidation may be a key player for initiating and/or mediating aspects of the aging process. Some work suggests that persons who live to exceptional ages, such as centenarians, might do so by aging more slowly [27]. If this is the case, then protection against lipid peroxidation may be a biological advantage that accounts, in part, for slower aging. 

The present work finds some support for this view. We confirmed that plasma LPO level increases with age in Okinawans, a very long-lived population, and drops off significantly by centenarian years. This is consistent with a survivor effect where those with high blood LPO levels are selected out (by death) leaving a population enriched with low blood LPO. Some protective endogenous and/or exogenous antioxidant defenses may be responsible for this effect. However, there is no clear indication from the present study that tocopherol and its subtypes (both plasma and intracellular) are leading biological factors in such a system, since blood tocopherol was lower in centenarians than in younger controls, with the possible exception of intracellular *β*-tocopherol. 

The fact that centenarians had higher intracellular *β*-tocopherol is interesting. While plasma *β*-tocopherol did not appear protective, some studies suggest that intracellular concentration of antioxidants might be more relevant than plasma levels since the source of most free radicals is intracellular mitochondria, and much damage occurs intracellularly—to DNA, cell membranes, and other cellular components. In addition, some data suggest that *β*-tocopherol might be a more effective antioxidant than *α*-tocopherol. For example, *β*-tocopherol is less likely to act as a free radical itself after quenching oxygen radicals than *α*-tocopherol [9]. This increased stability could, theoretically, result in better overall protection against oxidative stress. Researchers in [28] found that increased *β*-tocopherol was associated with decreased risk for metabolic syndrome, a life shortening prediabetic condition that increases oxidative stress. Researchers in [29] found that replacement of *α*-tocopherol by *β*-tocopherol enhances resistance to oxidative stress in a cell culture study.

Since tocopherols are lipid soluble, it is also possible that very old persons, who generally have low blood lipids, display artificially low blood tocopherol levels since it is mainly lipid bound. For example, our past data show that Okinawan centenarians have significantly lower total cholesterol and cholesterol subfractions (e.g., LDL, HDL) than elderly controls in their seventies [30]. Therefore, in older persons, tocopherol: lipid ratios might better reflect physiologic levels than blood tocopherol levels, whether in plasma or intracellular compartments. Nevertheless, correcting for different lipid levels across age strata was unrevealing. We did not find any significant differences between age groups for tocopherol: cholesterol and tocopherol: LPO ratios in centenarians versus younger controls.

Interestingly, a weakly negative correlation was found between intracellular tocopherol (T-tocopherol and *α*-tocopherol) and LPO in septuagenarians. Since mitochondria produce most of the oxidative stress in cells, higher intracellular levels of tocopherol should result in lower LPO levels and theoretically, less damage. However, this finding was only significant in septuagenarians and not in centenarians, and if this truly represents a protective factor for survival it is unclear why centenarians would not also exhibit this finding. More study of this issue is warranted.

Overall, the literature on oxidative stress as a longevity factor in humans is sparse. Some data from studies of Italian centenarians are in agreement with our LPO findings in Okinawan centenarians. For example, a study of 15 centenarians (3 males; 12 females) recruited from central Italy compared with equal numbers of controls (young, middle aged and elderly) showed that blood LPO levels, evaluated as MDA content, were lower in centenarians than in elderly control subjects (60–79 years of age) [31]. A larger study by the same research group also showed that LPO increased with age until centenarian years where a significant decrement occurred [32]. Centenarian LPO levels were lower than elderly controls (60–79 years) in that study as well but higher than young controls (aged 21–40 years). This suggests that centenarians might have mechanisms to keep oxidative stress at levels equivalent to persons who are decades younger, and this could help explain their exceptional longevity. Susceptibility to peroxidation of the erythrocyte membranes (when challenged) was also lower in centenarians than in older controls [32]. Vitamin E was not measured in these studies but another potential mechanism was identified. Centenarians were found to have increased levels of omega-3 polyunsaturated fatty acids (PUFAs) and reduced content of omega-6 PUFAs relative to younger controls. Long-chain omega-3 PUFAs decrease the production of inflammatory eicosanoids, cytokines, and reactive oxygen species by acting directly to inhibit arachidonic acid metabolism and acting indirectly to modify inflammatory gene expression [33].

While there were tentative links established in the current study between oxidative stress and vitamin E in the Okinawans, we were not able to strongly link vitamin E-derived tocopherols to reduced oxidative stress across all measures. LPO was higher in those aged in their 20s, 30s, and 70s and dropped off in the 80s and 100s. Most measures of vitamin E in this study also increased with age until the 70s and then dropped off. Similar trends have been seen in studies of younger, middle-aged, and older persons, although few studies included exceptionally old persons [8, 34–36]. The literature is mixed with regard to gender differences, with reports of higher plasma *α*-tocopherol in males [37] or females [38]. The current study found that females tended to have significantly higher tocopherol (total and *α*) levels than males. 

The lack of robust support in the current study and the literature in general for a powerful effect of vitamin E against LPO does not mean that vitamin E is unimportant to healthy aging. It is possible that measuring oxidative stress through TBA may not be the optimal method to assess vitamin E's antioxidant effects. Nevertheless, a clinical trial of vitamin E supplementation of up to 2,000 iu/day for 8 weeks was also not supportive of *in vivo* antioxidative effects. This study showed no change on three different indices of lipid peroxide (urinary 4-hydroxynonenal and 2 different isoprostanes) [39]. 

Interestingly, studies that focused on other measures of systemic defenses against oxidative stress *have* shown that centenarians exhibited higher red blood cell glutathione reductase and catalase activities, which could result in lower oxidative stress [40]. Studies of Danish centenarians also found that glutathione reductase activity was higher in centenarian erythrocytes than in younger controls. This was also linked to healthy aging where centenarians with the highest cognitive and physical function tended to have the greatest enzyme activity [41]. These might be fertile areas to search for relations with vitamin E.

Genes that influence oxidative stress might also be fertile areas for investigation, although genetic alterations of model organisms, including yeast, rodents, worms, and drosophila, have yielded conflicting data. In the fruit fly (*Drosophila melanogaster*), overexpression of antioxidative enzymes through knockout models or other means can dramatically increase lifespan. Conversely, the absence of CuZn superoxide dismutase, a major antioxidant, increases sensitivity to oxidative damage and reduces lifespan by approximately 80% but genetic rescue of half the enzyme restores lifespan [42, 43]. However, such dramatic effects are not seen in rodent models where mice with genetic alterations in antioxidant defenses typically show little alteration in lifespan, but do show alterations in healthspan [44]. For example, ApoE null mice have lower serum apolipoprotein E and develop significant early atherosclerosis [45] but overexpression of catalase, an important antioxidative defense, appears to confer protection against early atherosclerosis [46]. 

Interestingly, the two human genes that appear to have the strongest links to healthy aging and longevity, ApoE and FOXO3A [47–49] are both linked to oxidative stress. APOE genotype influences oxidative stress and inflammation in cell lines, rodents, and humans [50], and FOXO3A protects cells from oxidative stress [51] 

It is also possible that vitamin-E derived tocopherols may act through mechanisms that are not clearly linked to oxidative stress, such as second messenger molecular signaling [52] and/or other cardioprotective mechanisms [53], or membrane fluidity [54]. In addition, high vitamin E consumption may merely reflect an overall dietary pattern that results in healthier aging. For example, work on Italian centenarians shows that they possess relatively high plasma vitamin E [55]. Moreover, the traditional healthy Okinawan diet, which is associated with a high prevalence of centenarians, has 16.6 mg a day of vitamin E, which is a remarkable 190% of the Japan RDA. This is markedly higher than the Japanese traditional diet with 6.3 mg a day and 72% of the RDA for Japan (RDAJ = males: 10 mg/d; females: 8 mg/d) [15]. A study of Polish centenarians also showed that centenarians had high vitamin E levels compared with healthy, young female adults [40]. However, there has been no *causative *link to longevity.

Along the same line of thinking, many epidemiologic studies show a protective *association* for vitamin E with age-related chronic diseases but support from interventional clinical trials has been lacking [56]. For example, in a longitudinal study of healthy oldest-old (ages 80-plus), low plasma concentration of vitamin E and high concentration of lipid peroxide at baseline predicted risk of cardiovascular events over a 4-year follow-up [12]. While adjustment for vitamin C, *β*-carotene, and serum lipids did not affect the relation, the possibility still exists that other factors correlated with vitamin E could have been responsible for the association. In fact, a cross-sectional epidemiologic study of over 700 men and women, aged 25–74, showed that plasma LPO was positively correlated with a great many cardiovascular risk factors, including triglycerides, cholesterol subtypes, BMI, fibrinogen, and WBC count, and was negatively correlated with vitamin C [11]. It is difficult to control for all such associations in epidemiologic work, and the possibility that vitamin E is simply correlated with another more active molecule(s) cannot be completely ruled out. Indeed, when clinical trials have been conducted with vitamin E on incidence and progression of age-related chronic diseases the bulk of the evidence is not supportive of an important protective effect [56].

It is likely that several mechanisms account for low LPO at exceptional ages. Exceptional longevity has been linked to lower caloric intake, more efficient metabolism, increased insulin sensitivity, among other factors that might lead to lower oxidative stress [1, 14]. We have also found genetic factors that may be important, such as variants in HLA genes that may lead to less inflammation [57] and genetic variants in the FOXO3A gene with potentially pleiotropic mechanisms for reducing oxidative stress [48]. 

Finally, if there is truly less ongoing oxidative damage in centenarians then the etiology is probably complex and involves interplay of genetic and environmental factors that operate over a lifetime. A combination of scientific approaches will be needed to tease out the important mechanisms.

## Figures and Tables

**Table 1:** Plasma lipid peroxide in young, middle-aged, and older age groups (mean and SD), nmol/ml. LPO levels were measured using the TBA method [21, 22].

	20s	30s	70s	80s	100s
Male (*n*)	3.34 ± 1.79 (4)	4.06 ± 1.24 (8)***	3.15 ± 0.70 (11)***	2.92 ± 0.32 (5)***	1.49 ± 0.51 (30)
Female (*n*)	3.18 ± 0.64 (4)*	2.95 ± 0.53 (8)**	3.56 ± 0.81 (18)***	2.90 ± 0.46 (3)	1.72 ± 1.28 (109)
Total (*n*)	3.30 ± 1.25 (8)***	3.51 ± 1.08 (16)***	3.40 ± 0.79 (29)***	2.91 ± 0.34 (8)***	1.67 ± 1.16 (139)

Significant difference between centenarians and particular age group: *P < .05, **P < .01, ***P < .001.

**Table 2:** Mean plasma tocopherol (total and subtype) by age group (*μ*g/ml).

	Age	20s	30s	70s	80s	100s
*α* Toc	Male (*n*)	11.81 ± 3.03 (4)**	12.35 ± 3.60 (8)**	13.15 ± 7.82 (17)*	9.44 ± 1.07 (5)*	7.79 ± 2.10 (19)
	Female (*n*)	9.58 ± 2.62 (4)	11.07 ± 3.91 (8)	16.31 ± 11.64 (30)**	13.58 ± 3.63 (3)	9.82 ± 3.35 (72)
	Total (*n*)	10.69 ± 2.88 (8)**	11.71 ± 3.69 (16)**	15.14 ± 11.41 (47)***	11.28 ± 3.20 (8)*	9.37 ± 3.32 (91)

*β* Toc	Male (*n*)	0.25 ± 0.04 (4)	0.22 ± 0.05 (8)	0.23 ± 0.08 (17)*	0.20 ± 0.05 (5)	0.18 ± 0.07 (18)
	Female (*n*)	0.22 ± 0.04 (4)	0.21 ± 0.04 (8)	0.31 ± 0.18 (30)**	0.28 ± 0.13 (3)	0.20 ± 0.21 (69)
	Total (*n*)	0.23 ± 0.04 (8)	0.21 ± 0.04 (16)	0.28 ± 0.16 (47)**	0.23 ± 0.09 (8)	0.20 ± 0.19 (87)

*γ* Toc	Male (*n*)	1.37 ± 0.58 (4)	1.17 ± 0.40 (8)	1.00 ± 0.51 (17)	1.18 ± 0.33 (5)	0.93 ± 0.42 (18)
	Female (*n*)	0.98 ± 0.22 (4)	1.04 ± 0.50 (8)	1.62 ± 1.76 (30)*	1.16 ± 0.17 (3)	1.03 ± 0.59 (72)
	Total (*n*)	1.17 ± 0.46 (8)	1.11 ± 0.44 (16)	1.39 ± 1.45 (47)*	1.17 ± 0.49 (8)	1.01 ± 0.52 (90)

Total Toc	Male (*n*)	13.42 ± 3.60 (4)**	13.74 ± 3.70 (8)***	14.38 ± 8.28 (17)*	10.82 ± 1.37 (5)*	8.89 ± 2.31 (19)
	Female (*n*)	10.78 ± 2.80 (4)***	12.33 ± 3.87 (8)	18.24 ± 12.69 (30)***	15.01 ± 3.62 (3)***	11.05 ± 3.41 (91)
	Total (*n*)	12.10 ± 3.31 (8)***	13.03 ± 3.73 (16)***	16.81 ± 11.32 (47)***	12.68 ± 3.27 (8)***	10.58 ± 3.32 (110)

Toc: Tocopherol.

Significant difference between centenarians and particular age group: *P < .05; **P < 0.01; ***P < .001.

Significant difference between male and female centenarians: *P* < .01 in *α* Toc; *P* < .05 in total Toc.

**Table 3:** Mean intracellular (red blood cell) tocopherol levels by age strata (total and subtype) (*μ*g/ml).

		20s	30s	70s	80s	100s
*α* Toc	Male (*n*)	2.47 ± 0.49 (4)**	2.43 ± 0.35 (8)**	2.48 ± 0.25 (17)***	2.46 ± 0.28 (5)*	1.51 ± 0.84 (19)
	Female (*n*)	2.53 ± 0.35 (4)	2.79 ± 0.82 (8)	2.61 ± 0.76 (30)***	2.91 ± 0.87 (3)	2.00 ± 1.13 (77)
	Total (*n*)	2.50 ± 0.40 (8)**	2.61 ± 0.64 (16)**	2.56 ± 0.62 (47)***	2.66 ± 0.61 (8)*	1.89 ± 1.09 (96)

*β* Toc	Male (*n*)	0.05 ± 0.00 (4)	0.05 ± 0.01 (8)	0.04 ± 0.01 (17)*	0.05 ± 0.01 (5)	0.08 ± 0.04 (10)
	Female (*n*)	0.04 ± 0.00 (4)	0.04 ± 0.01 (8)	0.05 ± 0.02 (30)**	0.06 ± 0.02 (3)	0.10 ± 0.08 (37)
	Total (*n*)	0.05 ± 0.01 (8)	0.04 ± 0.01 (16)	0.05 ± 0.02 (47)**	0.05 ± 0.01 (8)	0.09 ± 0.07 (47)

*γ* Toc	Male (*n*)	0.35 ± 0.06 (4)	0.31 ± 0.12 (8)	0.27 ± 0.09 (17)	0.39 ± 0.10 (5)	0.25 ± 0.16 (18)
	Female (*n*)	0.35 ± 0.08 (4)	0.32 ± 0.11 (8)	0.33 ± 0.17 (30)*	0.31 ± 0.16 (3)	0.23 ± 0.19 (74)
	Total (*n*)	0.35 ± 0.07 (8)	0.32 ± 0.11 (16)	0.31 ± 0.15 (47)*	0.35 ± 0.13 (8)	0.24 ± 0.18 (92)

Total Toc	Male (*n*)	2.87 ± 0.50 (4)***	2.76 ± 0.41 (8)***	2.79 ± 0.30 (17)***	2.89 ± 0.37 (5)*	1.80 ± 1.00 (19)
	Female (*n*)	2.92 ± 0.39 (4)***	3.15 ± 0.75 (8)	2.98 ± 0.82 (30)***	3.24 ± 0.76 (3)***	2.30 ± 1.22 (77)
	Total (*n*)	2.90 ± 0.42 (8)***	2.95 ± 0.62 (16)***	2.91 ± 0.67 (47)***	3.05 ± 0.57 (8)***	2.19 ± 1.19 (96)

Toc: Tocopherol.

Significant difference centenarians and individual age groups: *P < .05; **P < 0.01; ***P < .001.

**Table 4:** Plasma and intracellular tocopherols adjusted by lipid subfraction.

		Septuagenarians (aged 70s)			Centenarians (aged 100s)		
		Male (*n*)	Female (*n*)	Total (*n*)	Male (*n*)	Female (*n*)	Total (*n*)

	T-Toc/Tc	0.08 ± 0.267 (17)	0.08 ± 0.259 (30)	0.08 ± 0.283 (47)	0.075 ± 0.018 (16)	0.072 ± 0.023 (68)	0.072 ± 0.021 (84)
Plasma	*α*-Toc/Tc	0.07 ± 0.252 (17)	0.08 ± 0.238 (30)	0.07 ± 0.285 (47)	0.06 ± 0.017 (15)	0.07 ± 0.018 (50)	0.07 ± 0.017 (65)

Intracellular	T-Toc/Tc	0.016 ± 0.010 (17)	0.013 ± 0.017 (30)	0.015 ± 0.017 (47)	0.015 ± 0.007 (16)	0.016 ± 0.010 (55)	0.016 ± 0.009 (71)
	*α*-Toc/Tc	0.014 ± 0.008 (17)	0.012 ± 0.016 (30)	0.013 ± 0.016 (47)	0.013 ± 0.006 (16)	0.014 ± 0.009 (55)	0.013 ± 0.009 (71)
	*γ*-Toc/Tc	0.005 ± 0.016 (17)	0.007 ± 0.036 (30)	0.007 ± 0.036 (47)	0.007 ± 0.003 (15)	0.007 ± 0.004 (50)	0.007 ± 0.004 (65)
	*β*-Toc/Tc	0.002 ± 0.003 (17)	0.002 ± 0.003 (30)	0.002 ± 0.004 (47)	0.002 ± 0.001 (16)	0.002 ± 0.002 (54)	0.002 ± 0.002 (70)

	T-Toc/HDL-C	0.27 ± 0.55 (17)	0.37 ± 0.98 (30)	0.33 ± 0.81 (47)	0.28 ± 0.13 (16)	0.27 ± 0.10 (69)	0.27 ± 0.11 (85)
Plasma	*α*-Toc/HDL-C	0.25 ± 0.52 (17)	0.33 ± 0.90 (30)	0.30 ± 0.82 (47)	0.25 ± 0.12 (15)	0.25 ± 0.09 (50)	0.25 ± 0.09 (65)
	*γ*-Toc/HDL-C	0.019 ± 0.03 (17)	0.033 ± 0.14 (30)	0.027 ± 0.10 (47)	0.03 ± 0.02 (15)	0.03 ± 0.02 (50)	0.03 ± 0.02 (65)

	T-Toc/HDL-C	0.052 ± 0.020 (17)	0.060 ± 0.063 (30)	0.057 ± 0.047 (47)	0.059 ± 0.037 (16)	0.056 ± 0.031 (55)	0.058 ± 0.032 (71)
Intracellular	*α*-Toc/HDL-C	0.047 ± 0.017 (17)	0.053 ± 0.058 (30)	0.050 ± 0.044 (47)	0.049 ± 0.032 (16)	0.049 ± 0.029 (55)	0.049 ± 0.030 (71)
	*γ*-Toc/HDL-C	0.005 ± 0.006 (17)	0.007 ± 0.013 (30)	0.006 ± 0.010 (47)	0.008 ± 0.006 (16)	0.007 ± 0.005 (54)	0.007 ± 0.006 (70)

Tc: Total cholesterol; HDL-C: high density lipoprotein cholesterol; Toc: Tocopherol. Note: no significant differences between groups.

**Table 5:** Average plasma tocopherol fractions/LPO across age strata.

Plasma Toc		20s	30s	70s	80s	100s
Total Toc/LPO	Male (*n*)	4.02 ± 2.01 (4)	3.38 ± 2.98 (8)	4.57 ± 11.83 (11)	3.71 ± 4.28 (5)	6.43 ± 3.61 (16)
	Female (*n*)	3.39 ± 4.38 (4)	4.18 ± 7.30 (8)	5.12 ± 15.67 (18)	5.18 ± 7.87 (3)	9.43 ± 14.22 (83)
	Total (*n*)	3.67 ± 2.65 (8)	3.71 ± 3.45 (16)	4.94 ± 14.33 (29)	4.36 ± 9.62 (8)	8.95 ± 13.13 (99)

*α* Toc/LPO	Male (*n*)	3.54 ± 1.69 (4)	3.04 ± 2.90 (8)	4.17 ± 11.17 (11)	3.23 ± 3.34 (5)	5.75 ± 3.29 (15)
	Female (*n*)	3.01 ± 4.09 (4)	3.75 ± 7.38 (8)	4.58 ± 14.37 (18)	4.68 ± 7.89 (3)	9.07 ± 13.86 (67)
	Total (*n*)	3.24 ± 2.30 (8)	3.34 ± 3.42 (16)	4.45 ± 13.18 (29)	3.88 ± 9.41 (8)	8.47 ± 12.70 (82)

*β* Toc/LPO	Male (*n*)	0.07 ± 0.02 (4)	0.05 ± 0.04 (8)	0.07 ± 0.11 (11)	0.07 ± 0.16 (5)	0.12 ± 0.07 (15)
	Female (*n*)	0.07 ± 0.06 (4)	0.07 ± 0.08 (8)	0.09 ± 0.22 (18)	0.10 ± 0.28 (3)	0.19 ± 0.24 (64)
	Total (*n*)	0.07 ± 0.03 (8)	0.06 ± 0.04 (16)	0.08 ± 0.20 (29)	0.08 ± 0.26 (8)	0.17 ± 0.26 (79)

*γ* Toc/LPO	Male (*n*)	0.41 ± 0.32 (4)	0.29 ± 0.32 (8)	0.32 ± 0.73 (11)	0.40 ± 1.03 (5)	0.63 ± 0.47 (15)
	Female (*n*)	0.31 ± 0.34 (4)	0.35 ± 0.948	0.46 ± 2.17 (18)	0.40 ± 1.54 (3)	1.06 ± 1.59 (67)
	Total (*n*)	0.36 ± 0.37 (8)	0.32 ± 0.41 (16)	0.41 ± 1.84 (29)	0.40 ± 1.44 (8)	0.98 ± 1.46 (82)

Toc: Tocopherol; LPO: lipid peroxidase. Note: no significant differences between groups.

**Table 6:** Intracellular tocopherol: lipid peroxide ratios across age strata.

Intracellular Toc		20s	30s	70s	80s	100s
Total Toc/LPO	Male (*n*)	0.86±0.28 (4)	0.68±0.33 (8)	0.89±0.43 (11)	0.99 ± 1.16 (5)	1.21 ± 1.12 (16)
	Female (*n*)	0.92 ± 0.61 (4)	1.07 ± 1.42 (8)	0.84±1.00 (18)	1.12 ± 1.65 (3)	1.67 ± 1.69 (71)
	Total (*n*)	0.88 ± 0.34 (8)	0.84 ± 0.57 (16)	0.86 ± 0.85 (29)	1.05 ± 1.68 (8)	1.58 ± 1.60 (87)

*α* Toc/LPO	Male (*n*)	0.74 ± 0.27 (4)	0.60 ± 0.28 (8)	0.79 ± 0.36 (11)	0.84 ± 0.88 (5)	1.02 ± 0.92 (16)
	Female (*n*)	0.80 ± 0.55 (4)	0.95 ± 1.55 (8)	0.73 ± 0.94 (18)	1.00 ± 1.89 (3)	1.40 ± 1.44 (71)
	Total (*n*)	0.76 ± 0.32 (8)	0.74 ± 0.59 (16)	0.75 ± 0.78 (29)	0.91 ± 1.79 (8)	1.33 ± 1.36 (87)

*β* Toc/LPO	Male (*n*)	0.01 ± 0.00 (4)	0.01 ± 0.01 (8)	0.01 ± 0.01 (11)	0.02 ± 0.03 (5)	0.06 ± 0.03 (8)
	Female (*n*)	0.01 ± 0.00 (4)	0.01 ± 0.02 (8)	0.01 ± 0.02 (18)	0.02 ± 0.04 (3)	0.12 ± 0.24 (34)
	Total (*n*)	0.02 ± 0.01 (8)	0.01 ± 0.01 (16)	0.01 ± 0.03 (29)	0.02 ± 0.03 (8)	0.11 ± 0.22 (42)

*γ* Toc/LPO	Male (*n*)	0.10 ± 0.03 (4)	0.08 ± 0.10 (8)	0.09 ± 0.13 (11)	0.13 ± 0.31 (5)	0.18 ± 0.20 (15)
	Female (*n*)	0.11 ± 0.13 (4)	0.11 ± 0.21 (8)	0.09 ± 0.21 (18)	0.11 ± 0.35 (3)	0.22 ± 0.21 (68)
	Total (*n*)	0.11 ± 0.06 (8)	0.09 ± 0.10 (16)	0.09 ± 0.19 (29)	0.12 ± 0.38 (8)	0.21 ± 0.21 (83)

Toc: Tocopherol; LPO: lipid peroxidase. Note: no significant differences between groups.

**Table 7:** Correlation coefficient between tocopherol and plasma lipid peroxide.

	Plasma tocopherol				RBC tocopherol			
	T-Toc	*α*-Toc	*β*-Toc	*γ*-Toc	T-Toc	*α*-Toc	*β*-Toc	*γ*-Toc

Centenarians (*n* = 139)	0.01	0.11	−0.03	−0.04	0.01	0.02	0.04	−0.06
Septuagenarians (*n* = 61)	0.34**	0.33**	−0.02	0.21	−0.32*	−0.29*	−0.16	−0.11

*P < .05, **P < .01.
